# Feasibility and effectiveness of a remote individual rehabilitation program for people with Parkinson's disease living in the Brazilian Amazon: a randomized clinical trial

**DOI:** 10.3389/fneur.2023.1244661

**Published:** 2023-08-25

**Authors:** Luciana Fernandes Pastana Ramos, Tamires de Cássia Santos Vilacorta-Pereira, Juliana dos Santos Duarte, Elizabeth Sumi Yamada, Bruno Lopes Santos-Lobato

**Affiliations:** ^1^Laboratório de Neuropatologia Experimental, Universidade Federal do Pará, Belém, PA, Brazil; ^2^Serviço de Neurologia, Hospital Ophir Loyola, Belém, PA, Brazil

**Keywords:** Parkinson's disease, telerehabilitation, physiotherapy, intervention, outcomes

## Abstract

Parkinson's disease (PD) is a chronic and progressive neurodegenerative disorder, and the current treatment involves pharmacological intervention and physiotherapy. Telerehabilitation, which involves remote support and guidance for patients undergoing rehabilitation, can potentially improve access to physiotherapy services for people with Parkinson's disease, especially those who face geographic barriers to healthcare. The primary aim of this study was to assess the feasibility and efficacy of a telerehabilitation program for people with Parkinson's disease living in an underrepresented community of the Brazilian Amazon. We conducted a parallel-group, single-center, single-blind, phase 2 randomized controlled clinical trial involving 19 participants diagnosed with Parkinson's disease from Belém, Brazil. Participants were assigned to a 4-week individual telerehabilitation program or a booklet-based exercise program (control group). Assessments were conducted before the intervention, immediately after the intervention, and 4 weeks after the end of the intervention. We showed that our telerehabilitation program had high adherence among patients, with minimal adverse effects. Both telerehabilitation and booklet orientation reduced the time to complete the Timed Up and Go test. In conclusion, our telerehabilitation program was feasible and effective for people with Parkinson's disease in an Amazonian setting. This trial was registered at the Registro Brasileiro de Ensaios Clínicos (ReBEC) under the identifier: RBR-6sz837s.

## 1. Introduction

The incidence of Parkinson's disease (PD) is escalating faster than any other neurological disorder (1). Nevertheless, resources and healthcare access for numerous individuals with PD remain insufficient as there are large inequalities across regions globally and among groups with different levels of income (2, 3).

In addition, the year 2020 marked the outbreak of the COVID-19 pandemic that compelled many countries to adopt stringent measures such as social distancing and service suspension, resulting in a significant impact on society and health. These measures caused a major shift in lifestyle, harming people's physical and mental wellbeing worldwide. The healthcare sector was severely affected, causing disruptions in treating several chronic illnesses, including PD (4, 5).

The available therapeutic approaches for PD are symptomatic and focus on alleviating motor and non-motor symptoms. Physiotherapy plays a crucial role in the clinical improvement of people with PD because the treatment based on exercise and physical activity has been suggested as an intervention that may lower the risk of developing PD and alter the disease progression through neuroprotective mechanisms (5–7).

Telerehabilitation is defined as the delivery of rehabilitation services at a distance utilizing videoconferencing through computers, tablets, or mobile phones equipped with integrated or external webcams (8). This technology enables remote connection between patients and rehabilitation professionals, aiming to enhance the wellness of individuals. In Brazil, the Federal Council of Physiotherapy and Occupational Therapy allowed telerehabilitation in response to the COVID-19 pandemic, avoiding the negative impact of social distancing (9).

Previous studies explored the feasibility and effect of telerehabilitation strategies for people with PD. Telerehabilitation in PD has been shown as a safe treatment, with high adherence and some improvement in motor symptoms, such as gait and balance (5, 10–12), including in Brazil (13, 14).

Studies on people with PD have neglected underrepresented populations, failing to address PD diagnosis and care in all communities (15). The recruitment of non-white populations for clinical trials remains limited, representing a significant obstacle to the progress of PD research, missing out on crucial genetic and molecular insights that may exist in other populations. Fortunately, a growing body of research on underrepresented communities started uncovering valuable information, emphasizing diversity in PD studies (16).

The Brazilian public healthcare system currently struggles to afford equal access to rehabilitation nationwide, with the lowest workforce of physiotherapy professionals in the Brazilian Amazon (17). Furthermore, the free movement of patients to have access to rehabilitation clinics through the Amazonian hinterlands is hampered by the fragile transport infrastructure and vast extensions of the region (18). For people with PD, this challenge is increased by progressive disability. Thus, the conventional face-to-face rehabilitation model for people with PD in the Brazilian Amazon may have low efficacy and high expenditure. There are no studies on this theme in the Amazonian context.

The present study aims to evaluate the feasibility and effectiveness of a telerehabilitation program for people with PD living in an underrepresented community of the Brazilian Amazon.

## 2. Methods

### 2.1. Study design and participants

This was a parallel-group, single-center, single-blinded, phase 2 randomized controlled trial to investigate the feasibility, safety, and efficacy of a telerehabilitation program in people with PD. This study was conducted following the Declaration of Helsinki, and the trial protocol was approved by the Ethical Committee of Hospital Ophir Loyola (CAAE: 42496620.9.0000.5550). Written informed consent was obtained from all participants before enrollment. This trial has been registered at Registro Brasileiro de Ensaios Clínicos (ReBEC) under the identifier: RBR-6sz837s.

From June 2021 to December 2022, people with PD diagnosed according to the United Kingdom Parkinson's Disease Society Brain Bank criteria (19) were screened from the Movement Disorders Unit of the Hospital Ophir Loyola (Belém, Brazil). The inclusion criteria were as follows: (i) age between 30 and 80 years, (ii) mild stage disease (Hoehn and Yahr stage equal to or less than 2), and (iii) stable antiparkinsonian medication dose in the previous month. The exclusion criteria were as follows: (i) secondary or atypical parkinsonism, (ii) severe psychotic symptoms (score >2 in item 1.2 of the International Parkinson's and Movement Disorder Society-Unified Parkinson's Disease Rating Scale), (iii) clinical diagnosis of dementia; (iv) diagnosis of severe systemic disease (such as infections, severe heart disease, malignant neoplasm, liver or kidney failure, and poorly controlled diabetes), (v) the presence of orthopedic diseases, other neurological diseases, or cardiac comorbidities that prevent or pose a risk for performing aerobic or stretching exercises, (vi) no access to teleconference technology (smartphone, tablet, or computer), and (vii) being unable to perform tasks on the computer or without family assistance, which is needed for remote activities.

Participants were randomly assigned (1:1 ratio) to two groups, namely, control and telerehabilitation, using a web-based system stratified for sex and disease duration. The randomization was carried out by a researcher not involved in patient recruitment or assessment or data analysis. The researchers involved in the patient assessment or data analysis were blinded to group allocation. Due to the nature of the intervention, participants and the physiotherapists who conducted the telerehabilitation sessions could not be blinded to the treatment allocation. Patients were instructed not to talk about the interventions during evaluations. Considering the exploratory aspect of the study, focused on feasibility, the sample size calculation was not performed.

### 2.2. Procedure

**Figure 1:**
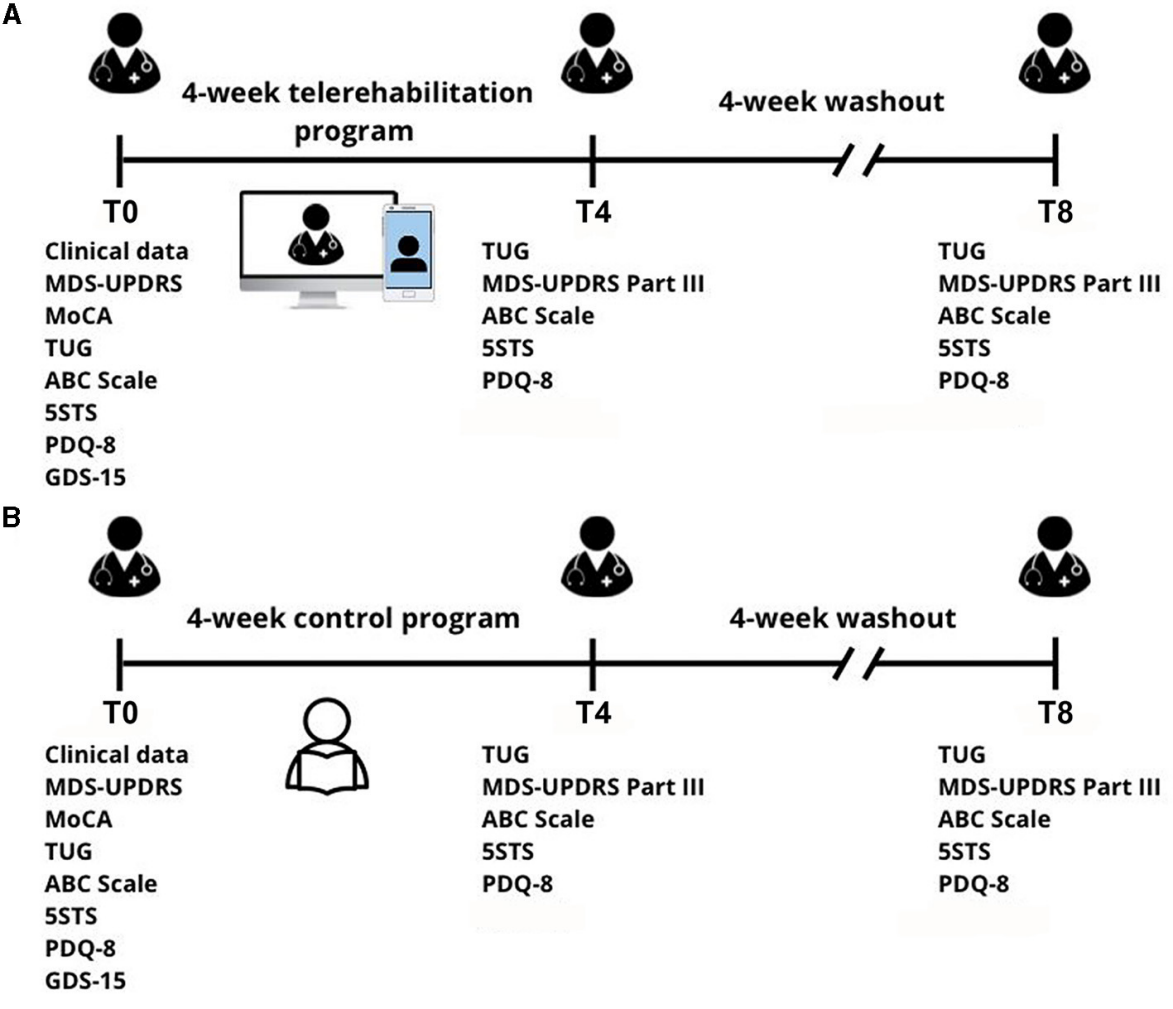
Study design. **(A)** Telerehabilitation (TR) group and **(B)** control (CT) group at the baseline (T0), end of intervention (T4), and follow-up at 8 weeks after the baseline (T8). 5STS, five-repetitions sit-to-stand test; ABC scale, activity-specific balance confidence scale; GDS-15, Geriatric Depression Scale; MDS-UPDRS, Movement Disorder Society-Unified Parkinson's Disease Rating Scale; PDQ-8, Parkinson's disease questionnaire-8; TUG, Time Up and Go Test.

Figure 1 presents a summary of the study schedule and assessment. Participants were evaluated at the baseline (T0), end of intervention (T4), and follow-up at 8 weeks after the baseline (T8) as wash-out evaluation. Participants were not allowed to receive any other type of rehabilitation during the study period. Since the randomization of participants, antiparkinsonian medications were not changed during the study period.

The telerehabilitation (TR) group underwent a 4-week telerehabilitation program at their homes, consisting of individual remote sessions supervised by a physiotherapist with real-time visual feedback and verbal cues (1 time/day, 3 days per week, for a total of 12 sessions, 60 min/session). Before sessions, participants' aerobic capacity and endurance were evaluated by the 6-min walk test (20). Only participants with good performance in the 6-min walk test would receive the exercise program. Telerehabilitation sessions were performed at home using a smartphone, tablet, or computer via free teleconference web platforms (e.g., Whatsapp^®^ or Google Meet^®^). A caregiver supervised all the sessions in person to assist the participants if needed.

Each session included warm-up, mobility, strength, balance, and cool-down exercises based on a previous therapist-supervised exercise protocol (21) of moderate level of intensity. In the first week, the telerehabilitation sessions focused on familiarizing the patients and their caregivers with the method and ensuring a clear understanding of the exercises. The physiotherapist verbally explained the exercises, and when patients faced difficulty executing them, the physiotherapist demonstrated the movements. In the second and third weeks, additional exercises were introduced to enhance postural control on unstable surfaces and facilitate the progression toward weight transfer while also considering eliminating visual input or including head movements. During the fourth week, dynamic balance exercises were incorporated to promote postural control while simultaneously involving movements of the upper and lower limbs. This phase aimed at enhancing the patients' overall dynamic stability and functional capabilities. The therapeutic interventions were carefully designed throughout the process to target proprioception in the foot, sacroiliac joint, and cervical spine, ensuring appropriate positioning during exercise sessions and fostering improvements in postural control and stability. If participants complained about fatigue during sessions, the level of intensity was reduced.

For the control (CT) group, the physiotherapist offered participants an informative illustrated booklet with a demonstration and description of the exercises from the telerehabilitation program. Participants were instructed to consult the booklet and perform the training three times per week. The booklet is available in Supplementary Appendix S1. For both groups, the physiotherapist checked training feedback and adverse effects (AEs) once a week via a telephone call.

At the baseline (T0), we collected demographic information, including age, gender, and educational status. Moreover, we evaluated clinical data, such as the Hoehn and Yahr stage (22), the Movement Disorder Society-Unified Parkinson's Disease Rating Scale (MDS-UPDRS) (23), the Montreal Cognitive Assessment (MoCA) (24), the Time Up and Go Test (TUG) (25), the five-repetitions sit-to-stand test (5STS) (26), the activities-specific balance confidence (ABC) scale (27), the Geriatric Depression Scale (GDS-15) (28), and the 8-item Parkinson's Disease Questionnaire (PDQ-8) (29).

At revaluations (T4 and T8), participants underwent only the following tests: MDS-UPDRS Part III, TUG, 5STS, ABC scale, and the PDQ-8.

### 2.3. Outcomes

The primary outcome was the feasibility of telerehabilitation sessions (TR group) and booklet-based exercise program (CT group). It was assessed by adherence and safety. Adherence was defined as the percentage of sessions attended. Based on the percentage of the sessions attended, participants were categorized as high adherence (>80%), partial adherence (20%−80%), and non-adherence (< 20%) (30). Safety was evaluated in both groups by tracking the cumulative number of AEs and severe AEs from the baseline through the end of follow-up. AEs were defined as all-cause mortality, hospitalization for falls, or other diseases preventing exercise participation. The occurrence of AEs was compared between groups.

As secondary outcomes, gait and dynamic movements were evaluated by the TUG test, 5STS, and ABC scale in T4 and T8. The global motor status was evaluated by MDS-UPDRS Part III. Patient-reported outcomes were evaluated by the PDQ-8.

### 2.4. Statistical analyses

The normality of data was checked with the Kolmogorov–Smirnov test. The results were reported as numbers and percentages for categorical variables and mean and 95% confidence interval (normal distribution) or median and interquartile range (non-normal distribution) for continuous variables. We used the two-tailed *t*-test (normal distribution) or Mann–Whitney test (non-normal distribution) to compare the characteristics of continuous data between groups at the baseline. The chi-square test was used to test binary data between the groups at the baseline.

We performed an intention-to-treat analysis. Changes from baseline to T4 and T8 in the secondary outcomes (TUG, 5STS, ABC scale, MDS-UPDRS Part III, and PDQ-8—normal distribution) were assessed using the two-way repeated-measure analysis of variance was applied using “Time” as the within-group factor and “Group” as the between-group factor, and the Bonferroni *post-hoc* test was used. Effect sizes were represented by the partial eta-squared values. The level of statistical significance for all tests was set at a *p*-value of < 0.05. SPSS (IBM SPSS Statistics, version 23.0) was used for statistical analyses.

## 3. Results

### 3.1. Study population and baseline characteristics

We invited 26 people with PD for the study. Nineteen participants (73% recruitment success) volunteered to participate and were randomized for the groups (CT group: *n* = 11; TR group: *n* = 8). All TR group participants had good performance in the 6-min walk test and completed 12 telerehabilitation sessions. One participant in the CT group did not complete the follow-up evaluation at T4. At T8, two participants of the CT group did not complete the follow-up evaluation, and one participant of the TR group did not complete the follow-up evaluation (CT group dropout rate 27%; TR group dropout rate 12.5%; and total participants dropout rate 21%).

**Figure 2:**
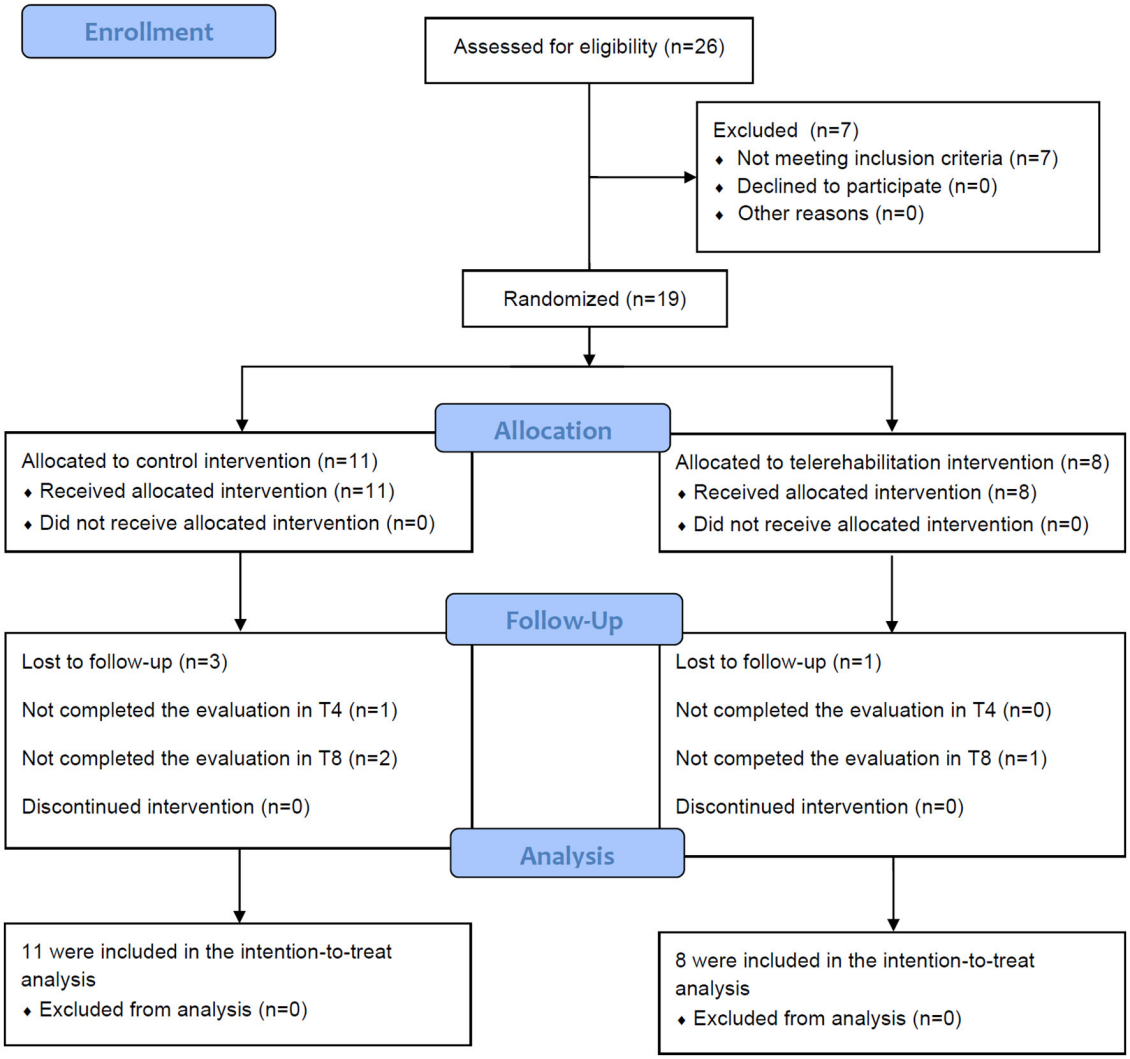
Study flowchart. *n*, number of participants; T4, evaluation at the end of intervention; T8, evaluation 8 weeks after the baseline.

A total of 18 participants (CT group: *n* = 10; TR group: *n* = 8) completed the T4 evaluation, and 15 (CT group: *n* = 8; TR group: *n* = 7) participants completed the T8 evaluation (Figure 2). Most participants were men (*n* = 10; 55.5%), aged from 47 to 75 years, and had a mean levodopa equivalent daily dose of 760 mg/day. Baseline clinical and demographic characteristics were similar across both groups, with no significant between-group differences (Table 1).

**Table 1:** Clinical and epidemiological data of participants at the baseline according to the intervention groups (*n* = 19).

**General characteristics**	**CT group (*n* = 11)**	**TR group (*n* = 8)**	**p-value**
Male sex, % (*n*)	60 (6)	50 (4)	0.67^a^
Age at the time of evaluation (years)^b^	58.6 (53–64)	60.7 (49–72)	0.88^c^
Age at onset of PD (years)^b^	51.8 (44–60)	54.8 (41–68)	0.9^c^
Diagnosis time (years)^d^	4 (2–11)	5 (3–9)	0.63^e^
Years of education^b^	10.3 (8–12)	12.5 (9–16)	0.14^c^
Body mass index^b^	26.7 (25–28)	25.2 (20–30)	0.46^c^
Levodopa equivalent daily dose (mg/day)^b^	747 (398–1,097)	750 (317–1,182)	0.85^c^
MDS-UPDRS part I^b^	9 (1–17)	10.4 (4–17)	0.44^c^
MDS-UPDRS part II^b^	9.6 (5–14)	14.1 (7–21)	0.18^c^
MDS-UPDRS part III^b^	27.4 (21–34)	22.1 (11–33)	0.32^c^
MDS-UPDRS part IV^d^	0 (0–6)	3 (0–9)	0.27^e^
MDS-UPDRS total score^b^	49.5 (32–67)	52.7 (31–74)	0.73^c^
**Hoehn and Yahr stage**			
Stage 1, % (*n*)	10 (1)	12.5 (1)	0.86^a^
Stage 2, % (*n*)	90 (9)	87.5 (7)	
Stage 3, 4, and 5 % (*n*)	0 (0)	0 (0)	
**GDS-15**			
No depression (0–5), % (*n*)	50 (5)	25 (2)	0.28^a^
Mild depression (6–10), % (*n*)	40 (4)	75 (6)	
Severe depression (+11), % (*n*)	10 (1)	0 (0)	
MoCA^d^	25 (22–25)	23 (16–24)	0.69^e^
TUG (s)^b^	17.4 (14–21)	17.3 (13–21)	0.69^c^
5STS (s)^b^	17.2 (14–20)	16.9 (11–23)	0.56^c^
ABC scale (%)^b^	63.9 (42–86)	67.6 (49–86)	0.96^c^
PDQ-8 (%)^b^	38.8 (20–57)	38.3 (17–60)	0.97^c^

^a^Chi-square test comparing frequencies between the booklet-based program (CT Group) and the telerehabilitation program (TR Group) at the baseline.

^b^Values in mean (95% confidence interval).

^c^T-test comparing mean between the booklet-based program (CT Group) and the telerehabilitation program (TR Group) at the baseline.

^d^Values in median (interquartile range).

^e^Mann–Whitney test comparing medians between the booklet-based program (CT Group) and the telerehabilitation program (TR Group) at the baseline.

5STS, five-repetitions sit-to-stand test; ABC scale, activity-specific balance confidence scale; GDS-15, Geriatric Depression Scale; MDS-UPDRS, Movement Disorder Society–Unified Parkinson's Disease Rating Scale; PDQ-8, Parkinson's disease questionnaire-8; TUG, Time Up and Go Test.

### 3.2. Feasibility

All participants randomized to the TR group completed 12 telerehabilitation sessions, with 100% of the number of participants. There were no deaths, hospitalizations, or life-threatening AEs in any group. No AEs occurred during the evaluations or any of the telerehabilitation sessions. Eleven participants (57.8%) reported at least one AE. The proportion of individuals who experienced some AEs was similar between the TR group compared to the CT group (75 vs. 45%, *p* = 0.35).

In the TR group, two participants reported flu symptoms, three others presented pain, and one individual presented tiredness. In the CT group, three participants reported flu symptoms, one reported pain, and one was diagnosed with COVID-19. For none of the individuals, these AEs were a reason to interrupt the intervention or result in the loss of follow-up. There were no reports of falls.

### 3.3. Effects of the interventions on the secondary outcomes

**Figure 3:**
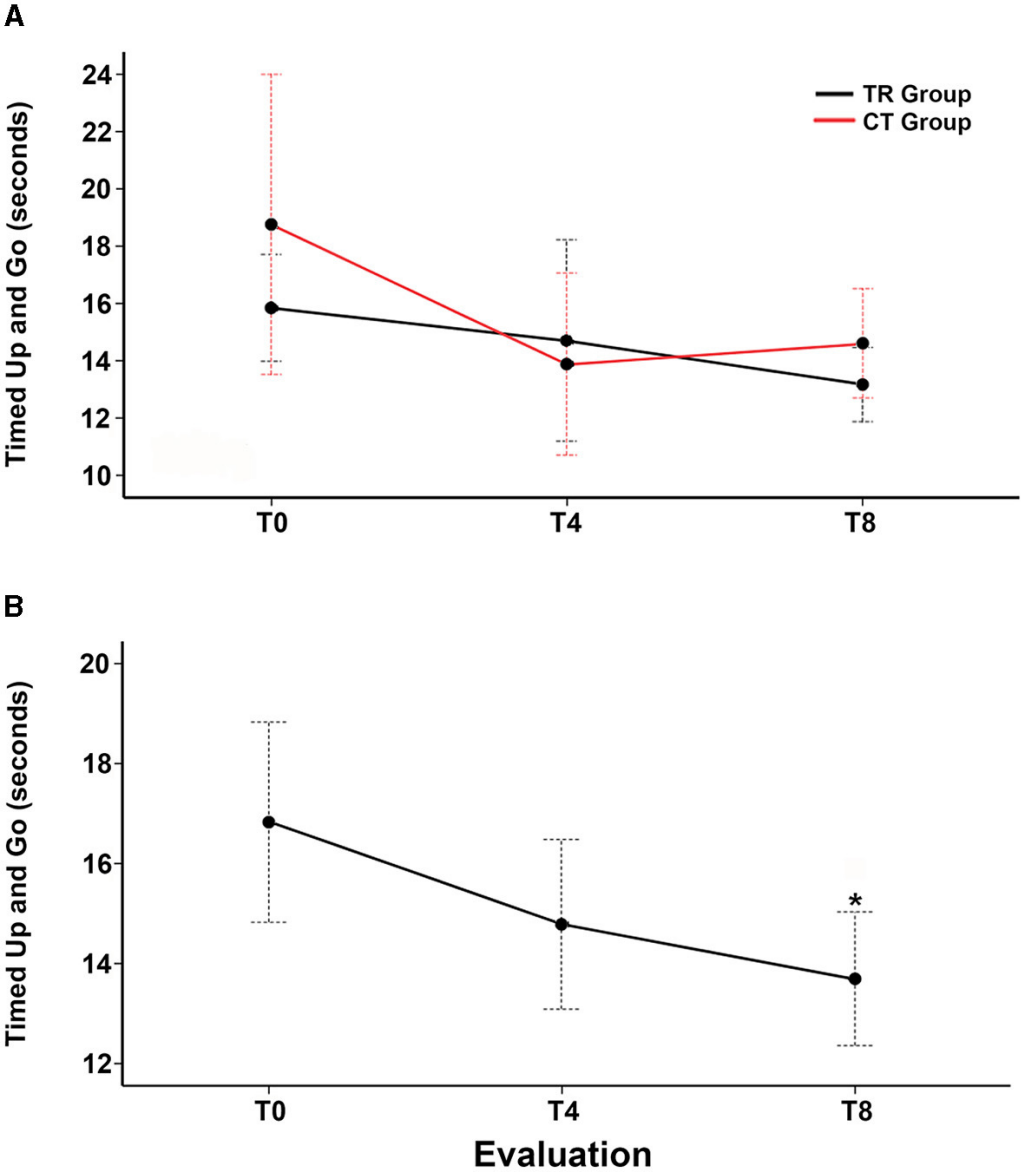
Change from the baseline in the Timed Up and Go test over 8 weeks. **(A)** Estimated marginal mean time to complete the Timed Up and Go test (in seconds) at the baseline (T0), post-intervention at 4 weeks (T4), and follow-up evaluation at 8 weeks (T8) in the telerehabilitation group (black line) and control group (red line). **(B)** Estimated marginal mean time to complete the Timed Up and Go test (in seconds) at T0, T4, and T8 of all participants. Red and black dotted I bars indicate standard errors.

Regarding TUG, there was no significant difference between the TR and CT groups in the T4 and T8 evaluations (Figure 3A; Table 2). When the two groups were analyzed together, there was a reduction in TUG in T8 compared to the baseline (*F*_2, 26_ = 7.47, *p* = 0.004; Bonferroni *post-hoc* test: *p* = 0.004), with a partial eta-squared of 0.36, indicating a large effect size (Figure 3B; Table 2).

**Table 2:** Secondary outcomes at the baseline (T0), end of intervention (T4), and follow-up at 8 weeks after the baseline (T8).

**Outcomes**	**Overall (*****n*** = **19)**				***p*-value 1**	**Partial eta-squared 1**	**CT group (*****n*** = **11)**			**TR group (*****n*** = **8)**			***p*-value 2**	**Partial eta-squared 2**
	**T0**	**T4**	**T8**	**Within-group change from the baseline after 8 weeks**			**T0**	**T4**	**T8**	**T0**	**T4**	**T8**		
TUG	16.8 (0.9)	14.7 (0.8)	13.6 (0.4)	3.41 (0.6)	0.004	0.36	18.7 (2.4)	13.8 (1.5)	14.5 (0.6)	15.8 (1.1)	14.6 (2.0)	13.1 (0.7)	0.35	0.28
5STS	16.1 (1.2)	14.7 (0.9)	13.7 (0.5)	2.05 (2.0)	0.07	0.2	17.2 (2.4)	14.3 (0.8)	13.9 (0.9)	14.7 (2.6)	15.7 (2.9)	13.9 (1.8)	0.25	0.4
ABC scale	66.9 (6.8)	71.3 (7.4)	68 (6.7)	2.95 (5.2)	0.52	0.04	77.8 (10.3)	67.6 (13.2)	64.3 (15.2)	69.8 (12.7)	75.9 (11.6)	77.4 (10.8)	0.13	0.53
PDQ-8	36.3 (6.2)	29.6 (4.8)	32.2 (4.6)	−0.26 (4.4)	0.21	0.11	30.4 (8.1)	36.7 (10.2)	37.2 (7.1)	27.3 (7.1)	19.5 (7.7)	21.0 (7.7)	0.28	0.35
MDS-UPDRS part III	24.2 (2.9)	21.1 (2.9)	15.6 (2.6)	7.87 (5.3)	0.07	0.18	30.2 (6.7)	28.5 (6.9)	17.5 (5.0)	18.2 (5.9)	16.7 (5.1)	15.2 (6.2)	0.55	0.14

Values in T0, T4, and T8 were presented as estimated marginal means and standard errors. Estimated marginal means, standard errors, p-values, and partial eta-squared values were calculated by the two-way repeated-measure analysis of variance. p-value 1 and partial eta-squared 1 are from the analysis with all participants, and p-value 2 and partial eta-squared 2 are from the analysis of the TR and CT groups.

5STS, five-repetitions sit-to-stand test; ABC scale, activity-specific balance confidence scale; CT Group, booklet-based program; MDS-UPDRS, Movement Disorder Society–Unified Parkinson's Disease Rating Scale; PDQ-8, Parkinson's disease questionnaire-8; TR Group, telerehabilitation program; TUG, Time Up and Go Test.

For 5STS, PDQ-8, and MDS-UPDRS Part III, we found that the results were not significant when compared between groups in T4 and T8 (Table 2).

## 4. Discussion

In this phase 2 clinical trial, we found that a 4-week individual home-based exercise program, administered through a telerehabilitation system, was feasible and safe for people with mild-to-moderate PD. Regarding secondary outcomes, no significant differences were observed between the telerehabilitation and control groups. Nevertheless, the overall analysis revealed that both telerehabilitation sessions and the booklet-based program reduced the total time to complete the TUG test after 8 weeks compared to baseline measurements when analyzed together.

A high level of adherence is fundamental for the proper development of telerehabilitation. Our study found a high adherence among participants of the telerehabilitation group. Other studies on people with PD incorporating telerehabilitation reported adherence rates of over 90% (14, 31). Moreover, studies with a lower adherence rate may impair power analysis (13).

Regarding AEs, both groups reported pain, which was predominantly mild and did not lead to discontinuation of the intervention. All reported AEs were minor events, aligned with the numbers and types of AEs described in traditional in-person rehabilitation programs (12, 14, 32, 33). In the present study, no falls were observed, addressing the primary concern regarding the physiotherapy component of the program.

Motor changes in PD are directly associated with an increased risk of falls (34). The TUG test is crucial for assessing functionality, gait, and balance in daily functional situations. Impaired TUG performance may also indicate subtle motor deficits and a potential prodromal marker for the risk of PD development. Subjects with longer TUG time (≥10 s) exhibited a higher risk of developing PD (35). A reduction of 3.63 s in TUG completion time after the tap test in normal pressure hydrocephalus was suggested as the minimal clinically important difference for the test (36).

Despite showing no differences between telerehabilitation and control groups in TUG time, there was a non-significant reduction in completion time at T4, with a significant difference at T8 in the overall analysis of our study. The positive impact of both telerehabilitation sessions and the booklet-based program in the TUG time persisted even 4 weeks after the interventions ended, suggesting a long-term and cumulative effect. The mean difference from T0 to T8 showed a reduction of 3.15 s close to the minimal clinically important difference previously suggested for the TUG test (36). Our results align with a prospective study that reported a significant reduction in TUG test time following a telehealth physical rehabilitation program (37).

Since the beginning of social isolation due to the COVID-19 pandemic, telerehabilitation services have been encouraged as a management strategy for people with PD due to their advantages, such as low cost and the absence of a need to travel to therapy, providing a concrete opportunity to improve access to rehabilitation (4, 5, 11, 14). In our study, technological problems did not affect adherence. Unlike other studies which required specific software and devices, our telerehabilitation program was based on common and free teleconference platforms for any available smartphone, tablet, or computer with minimum configurations, reducing access barriers for people with PD from any location.

Another advantage of home-based telerehabilitation is that treatment occurs in a familiar environment where the patient feels comfortable and safe, leading to beneficial results and positive outcomes for participants, especially in individuals with difficulties accessing hospital services. Although most telerehabilitation studies use synchronous, real-time rehabilitation, this study showed that including an asynchronous intervention (booklet) was also effective in people with PD. Torriani-Pasin et al. (14) reported that a video-based asynchronous remote physical exercise program is safe and considered an alternative to an in-person program for people with PD.

In a recent systematic review (38), the use of booklets as a fall self-management intervention for people with PD promoted a healthy lifestyle. Fall self-management booklets may inform patients about available resources, training to communicate with healthcare professionals, and common practical strategies to reduce fall risk.

There are a few limitations. The short duration of interventions (4 weeks) may not be adequate to measure adherence and safety for long-time treatments. The self-reported adherence rates and occurrence of AEs via telephone calls once a week in the control group might result in less reliable information about training feedback. Furthermore, a larger sample size of participants would be necessary to evaluate efficacy in motor and non-motor symptoms. Recruiting only participants with access to teleconference technology may cause a selection bias in the study.

## 5. Conclusion

Our 4-week individual telerehabilitation program in an underrepresented community of the Brazilian Amazon had a high adherence and low AEs. The telerehabilitation sessions and the booklet-based program reduced the time for the TUG test in people with PD. Therapeutic exercise implemented through home-based remote physiotherapy is a promising strategy for improving PD symptoms. Further studies with long-term synchronous telerehabilitation associated with asynchronous interventions in people with PD are needed.

## Data availability statement

The raw data supporting the conclusions of this article will be made available by the authors, without undue reservation.

## Ethics statement

The studies involving humans were approved by Ethical Committee of Hospital Ophir Loyola. The studies were conducted in accordance with the local legislation and institutional requirements. The participants provided their written informed consent to participate in this study.

## Author contributions

LFPR, TCSV-P, ESY, and BLS-L contributed to the conception and design of the study. LFPR, TCSV-P, JSD, and BLS-L contributed to the acquisition of data for the article. BLS-L organized the database. LFPR and BLS-L performed the statistical analysis. LFPR and TCSV-P wrote the first draft of the manuscript. LFPR, TCSV-P, and BLS-L wrote sections of the manuscript. All authors contributed to the manuscript revision, and read and approved the submitted version.

## Supplementary material

Supplementary Appendix S1 Booklet with demonstration and description of the exercises, in Portuguese. Each participant and caregiver allocated to the control group received a booklet and detailed orientations from the physiotherapist to perform the training three times per week.
