# Supplementary material for: Feasibility and effectiveness of a remote individual rehabilitation program for people with Parkinson's disease living in the Brazilian Amazon: a randomized clinical trial
Source: Front Neurol. 2023 Aug 25;14:1244661. doi: 10.3389/fneur.2023.1244661 (PMC10485362; doi:10.3389/fneur.2023.1244661)
Supplement: Supplementary Appendix S1 — Booklet with demonstration and description of the exercises, in Portuguese. Each participant and caregiver allocated to the control group received a booklet and detailed orientations from the physiotherapist to perform the training three times per week. [file Data_Sheet_1.PDF]

CARTILHA

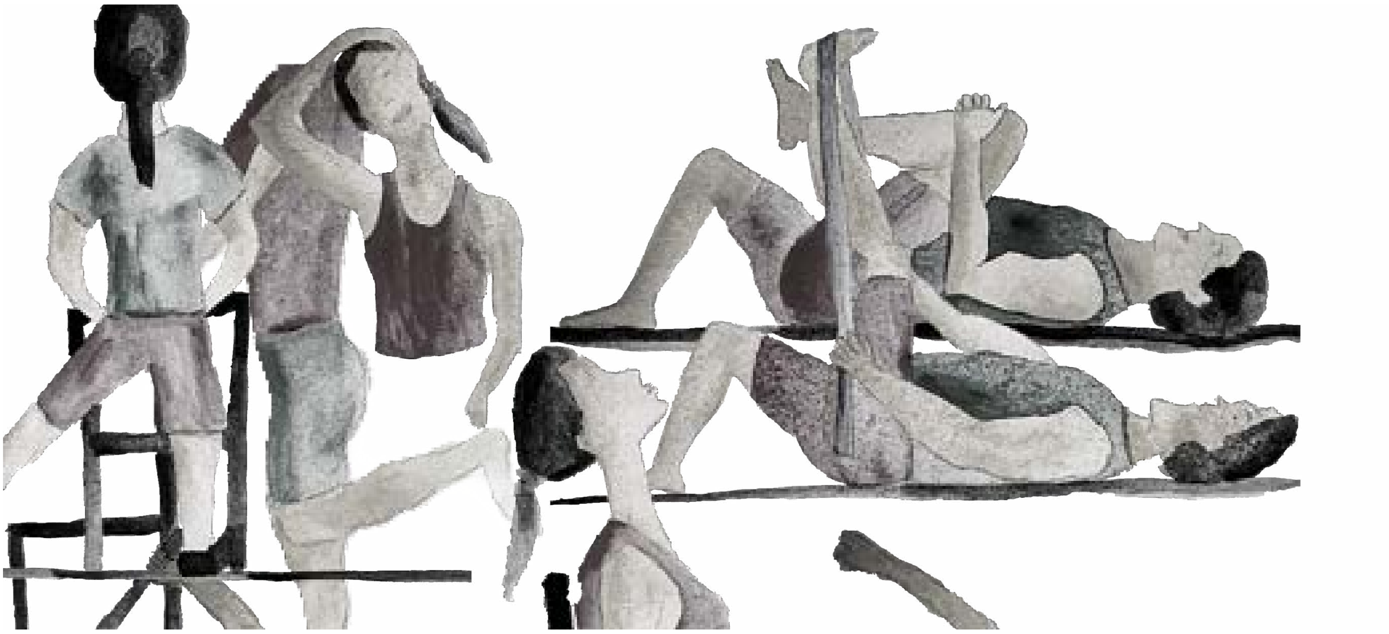

EXERCÍCIOS PARA FAZER EM CASA

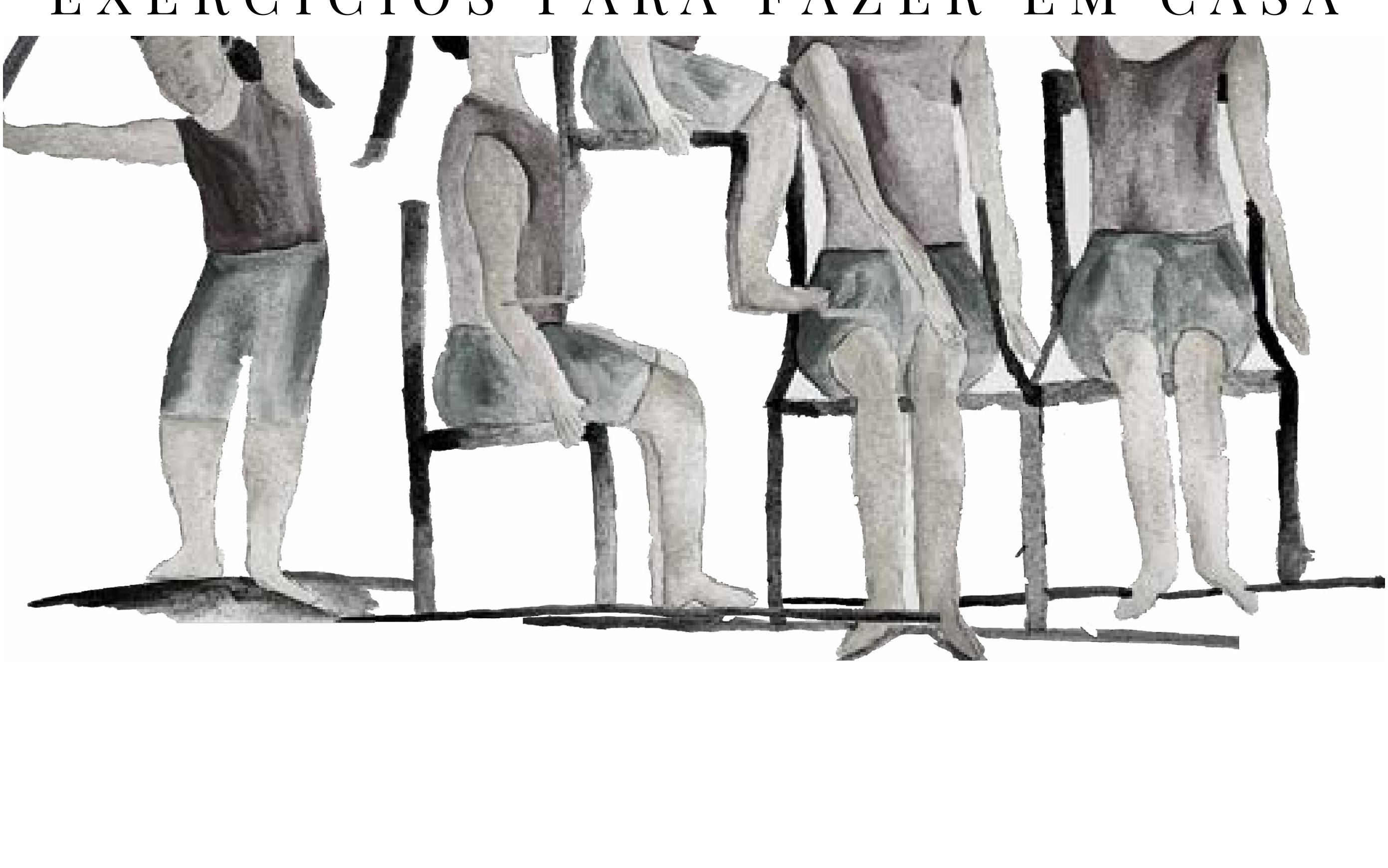

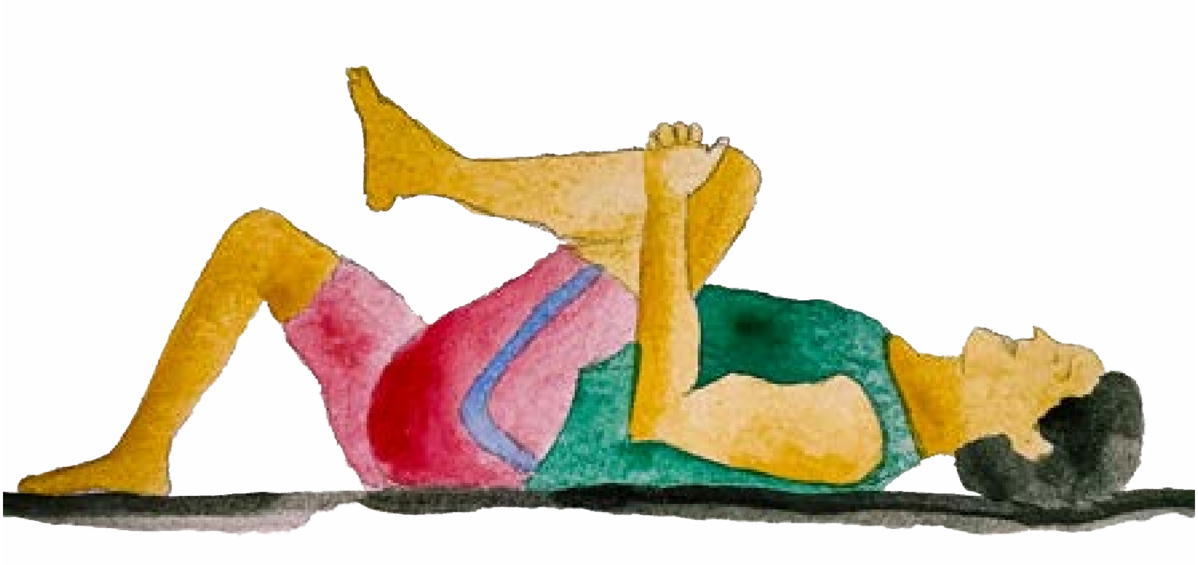

Vamos começar?

Deitado de barriga para cima

Puxe uma perna, colocando as mãos abaixo do joelho (igual você vê na figura acima)

Mantenha a posição por 20 segundos, respirando pelo nariz e soltando o ar pela boca

Depois repita o mesmo processo com a outra perna

Faça 3 vezes em cada perna

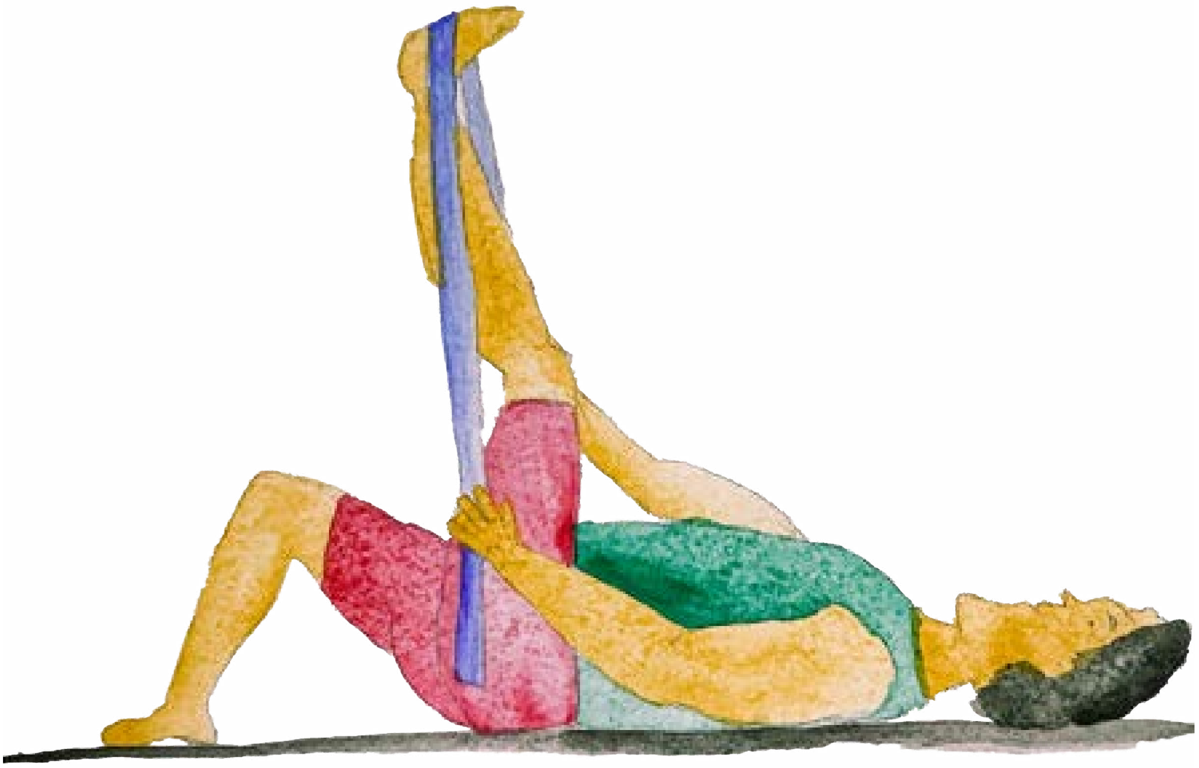

Deitado de barriga para cima

Estique a perna com o auxílio de uma tira de tecido (igual você vê na figura acima)

Mantenha a posição por 20 segundos, respirando pelo nariz e soltando o ar pela boca

Depois repita o mesmo processo com a outra perna

Faça 3 vezes em cada perna

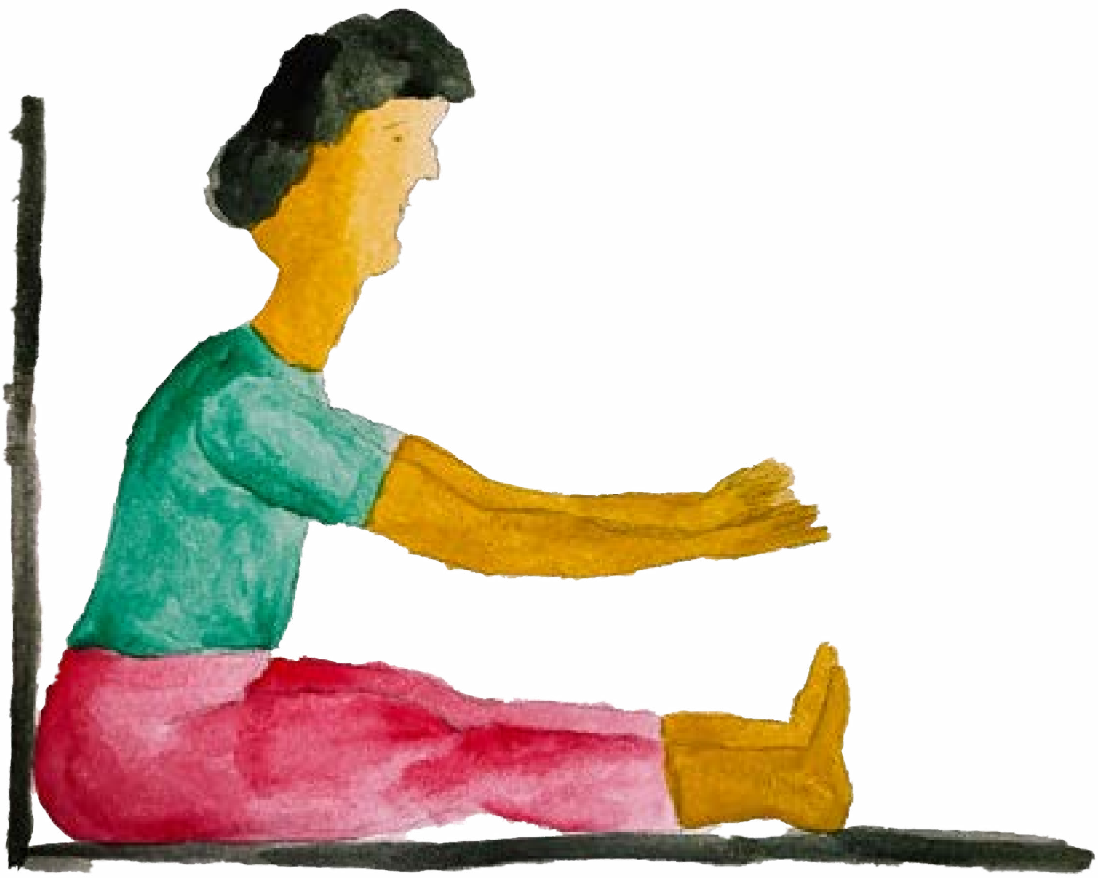

Sentado no chão

Tente manter a coluna reta

Estique os braços e leve as mãos tentando tocar nos pés (igual você vê na figura acima)

Faça o movimento de "ir e voltar" 5 vezes

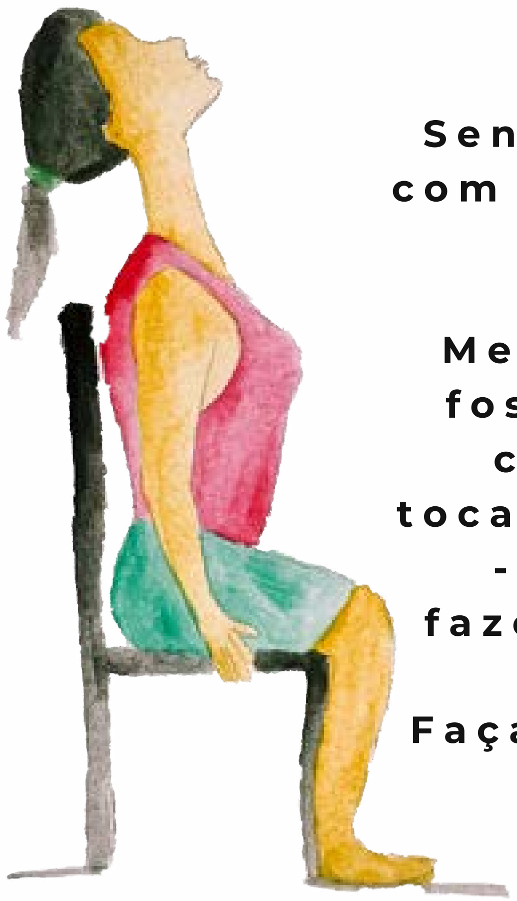

Sentado em uma cadeira com as costas apoiadas no encosto

Mexa a cabeça como se fosse olhar o teto ou o céu de pois retorne tocando o queixo no peito - como se estivesse fazendo o sinal de "sim"

Faça o movimento 3 vezes

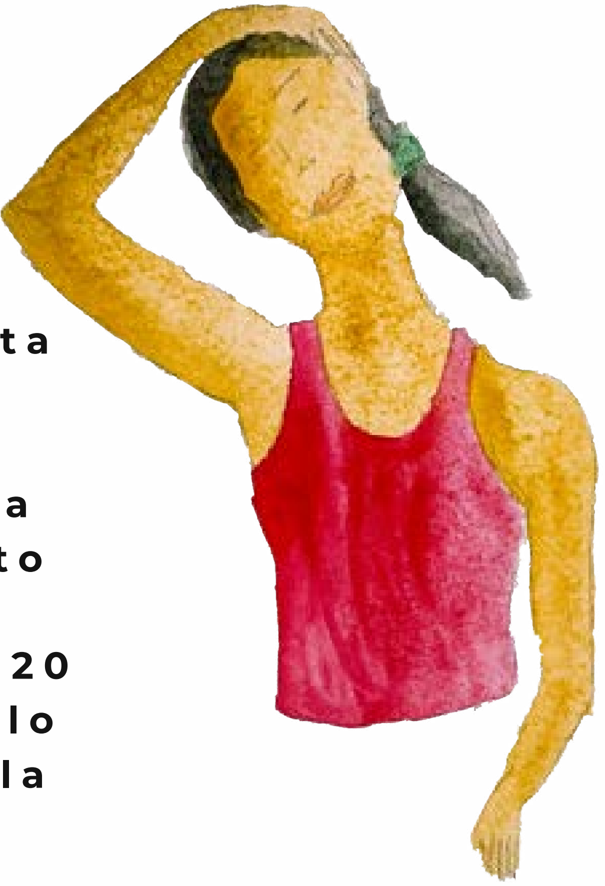

Ainda sentado em uma cadeira com as costas apoiadas no encosto
.
Coloque a sua mão direita posicionada acima da orelha esquerda, bem lentamente movimente a cabeça para o lado direito

Mantenha a posição por 20 segundos, respirando pelo nariz e soltando o ar pela boca

Depois repita o mesmo processo com a outra mão

Faça 3 vezes em cada lado

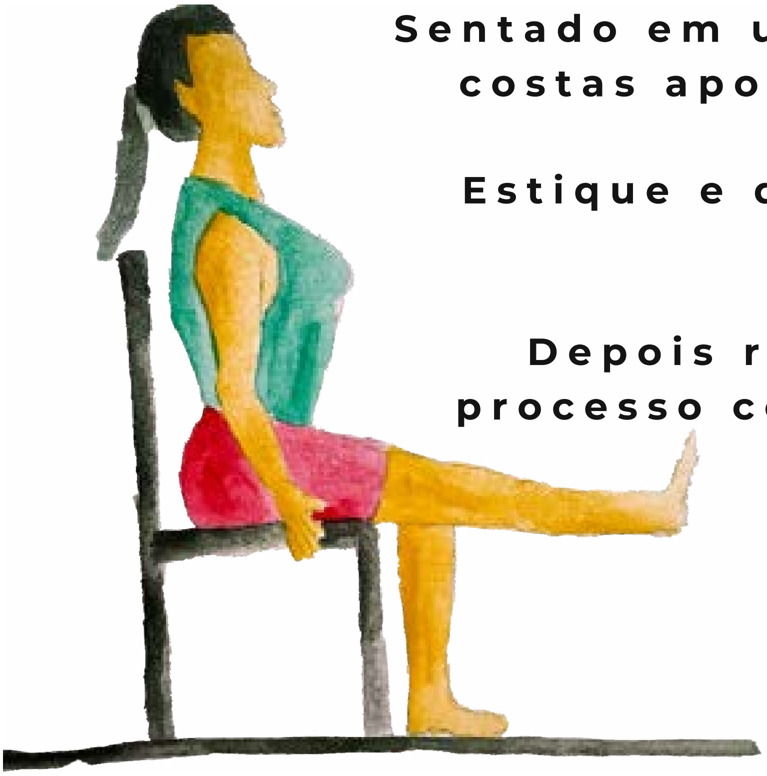

Sentado em uma cadeira com as costas apoiadas no encosto

Estique e dobre o joelho 10 vezes

Depois repita o mesmo processo com a outra perna

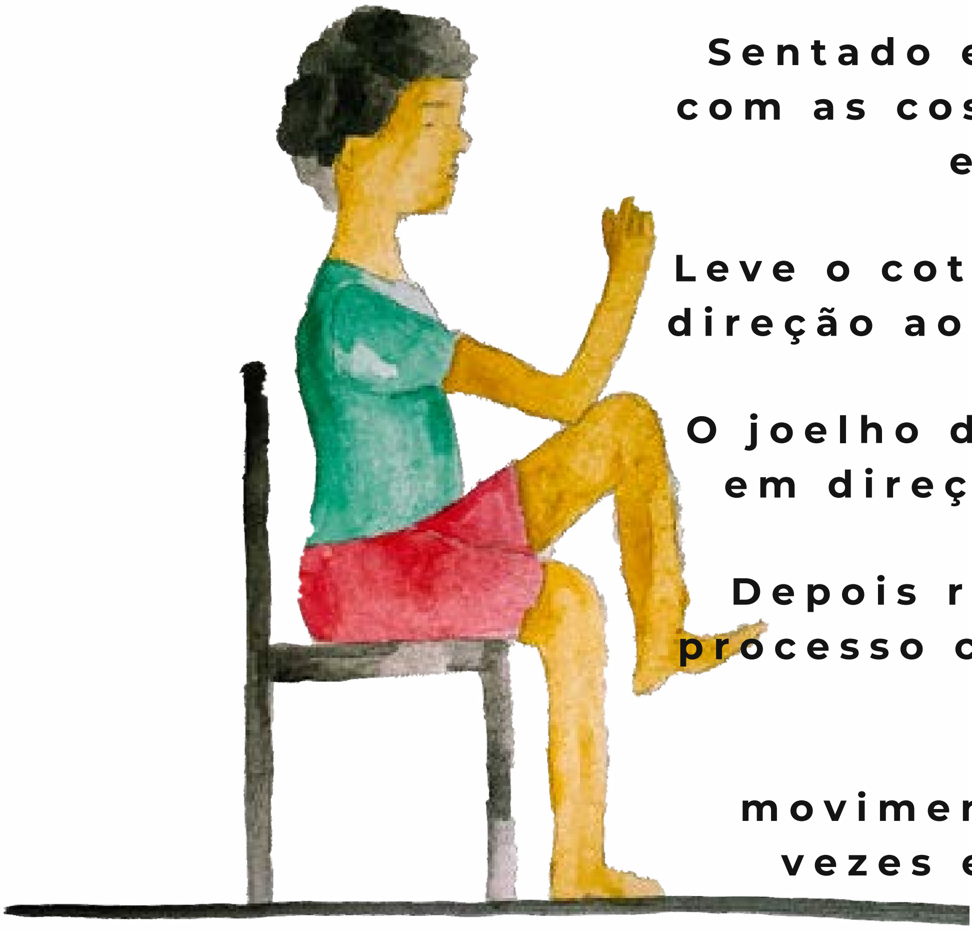

Sentado em uma cadeira com as costas apoiadas no encosto

Leve o cotovelo direito em direção ao joelho esquerdo

O joelho deve ser elevado em direção ao cotovelo

Depois repita o mesmo processo com o outro lado

Faça o movimento cruzado 10 vezes em cada lado

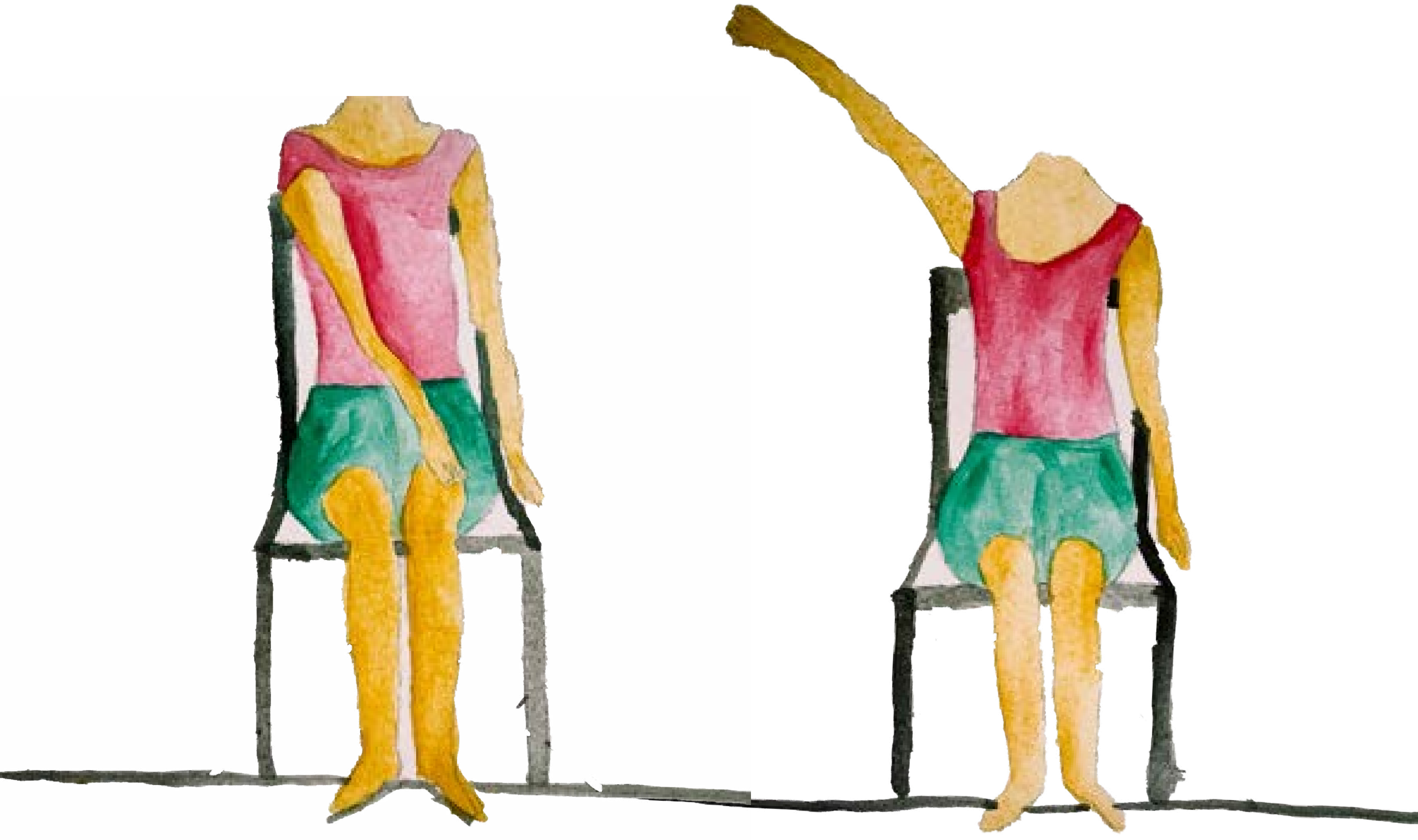

Sentado em uma cadeira com as costas apoiadas no encosto

Estique o braço e leve a mão direita em direção ao joelho esquerdo

Depois repita o mesmo processo com a outra mão

Faça o
movimento cruzado 10 vezes em cada lado

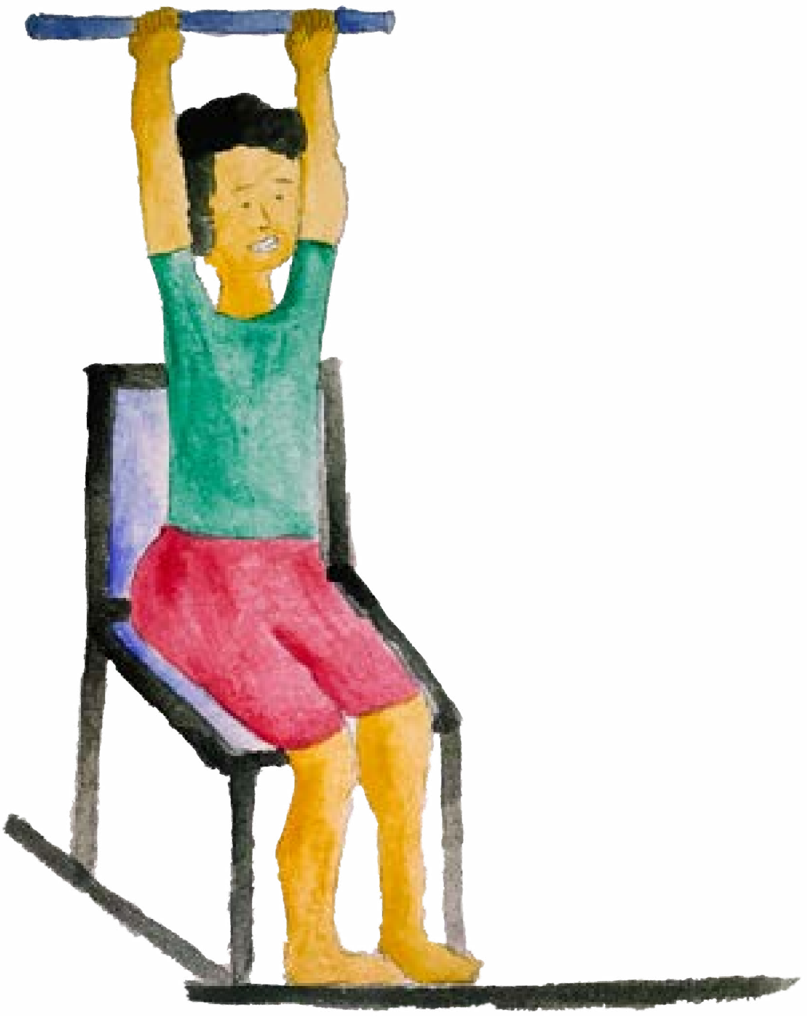

Pegue um cabo de vassoura

Sentado em uma cadeira com as costas apoiadas no encosto

Estique o braço segurando o cabo de vassoura com as duas mãos

Levante os braços até ficar em cima da cabeça e depois volte a tocar os joelhos

Faça o movimento 10 vezes

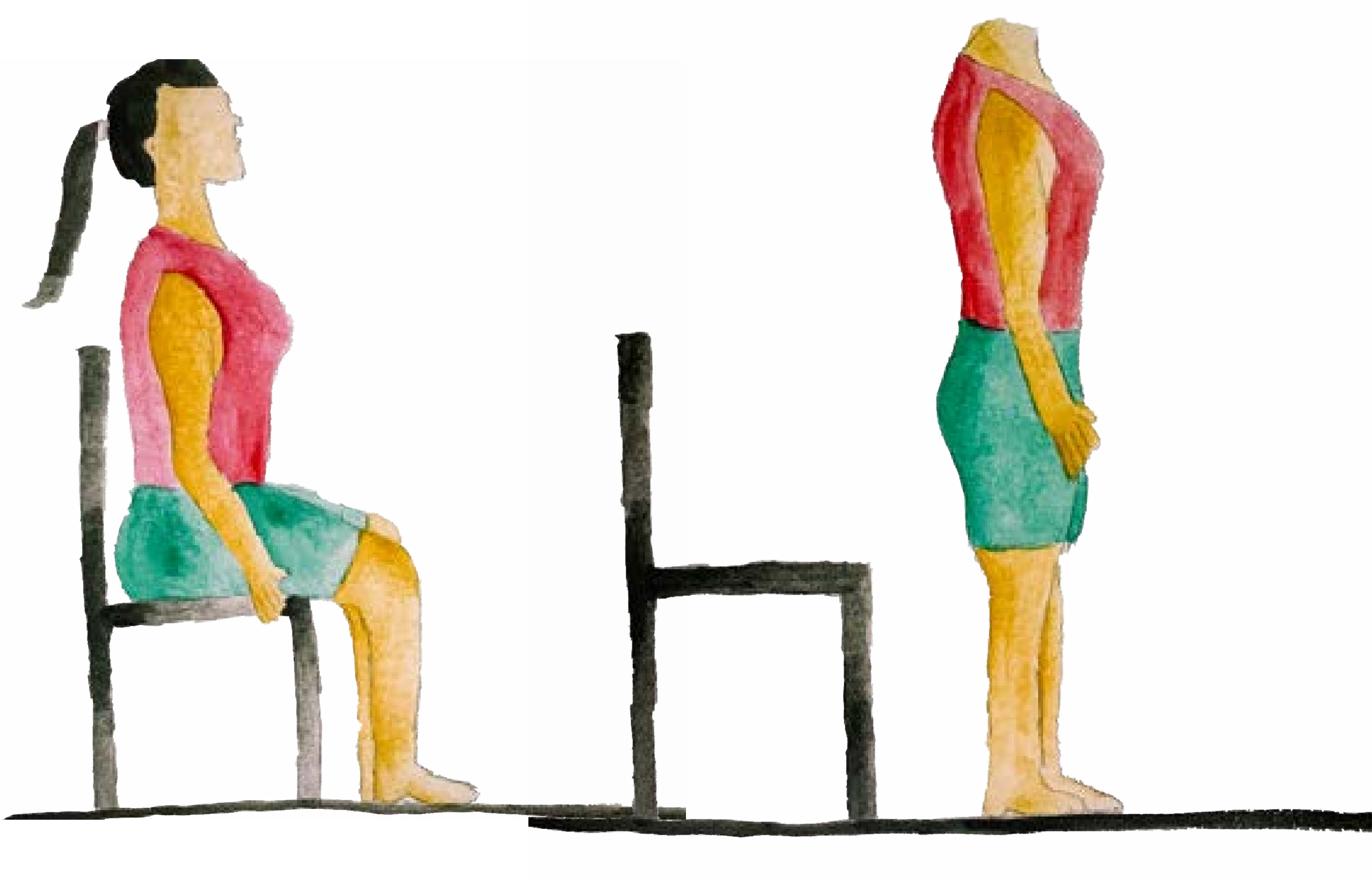

Levante e volte a sentar na cadeira

Faça o movimento 5 vezes

Descanse durante 1 minuto

Faça de novo o movimento 5 vezes

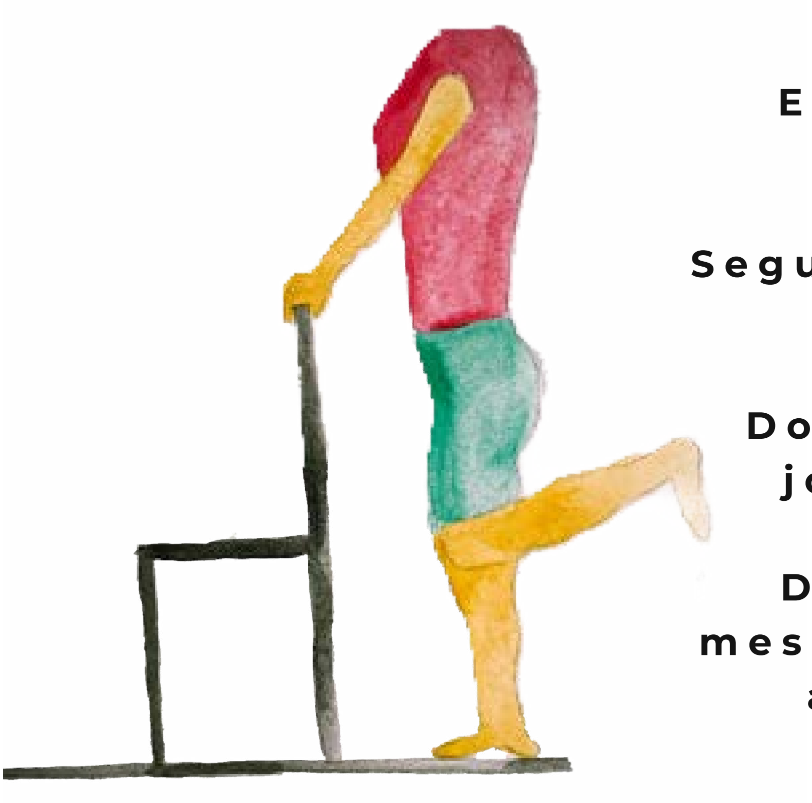

Em pé, atrás da cadeira

Segure no encosto da cadeira

Dobre e estique o joelho 10 vezes

Depois repita o mesmo processo com a outra perna

Em pé, atrás da cadeira

Segure no encosto da cadeira

Com o joelho esticado, leve os pés para trás e depois volte

Faça o movimento 10 vezes em cada perna

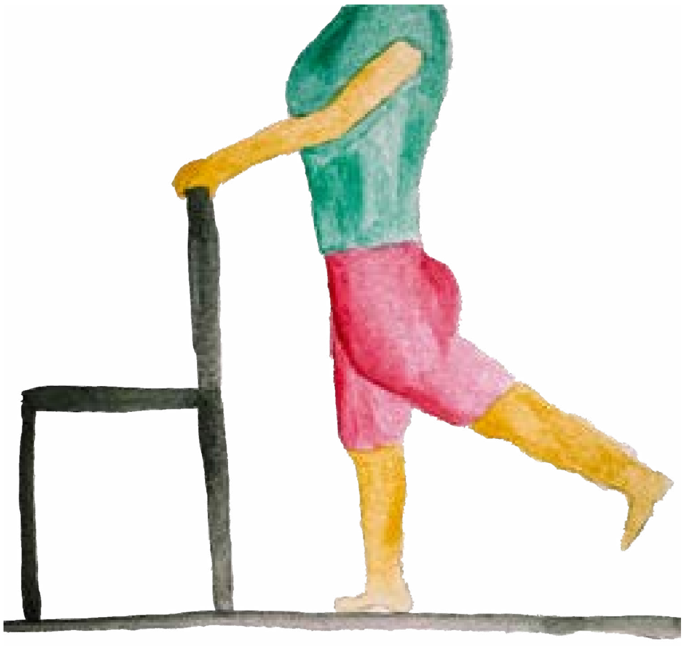

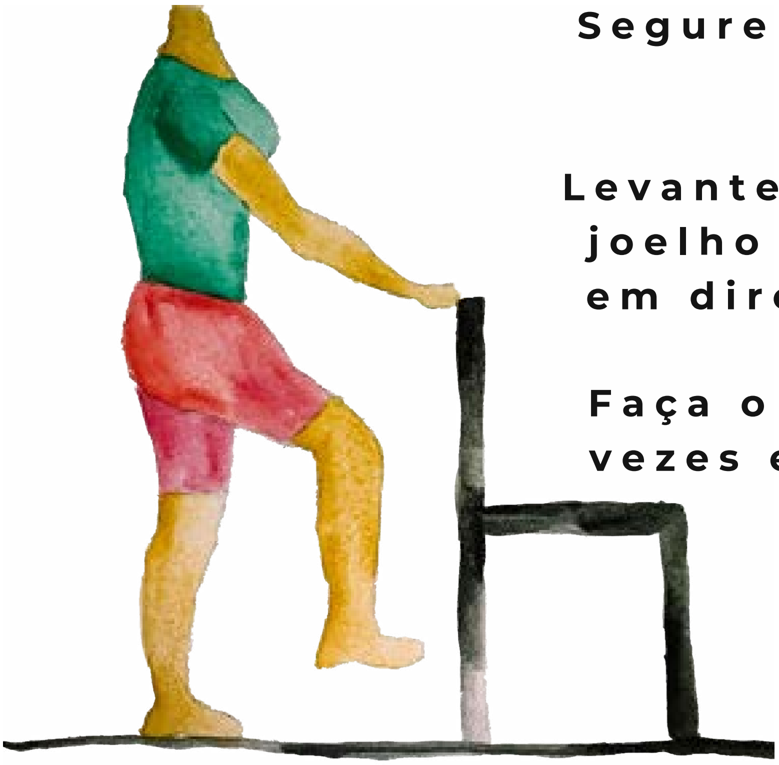

Em pé, atrás da cadeira

Segure no encosto da cadeira

Levante a perna com o joelho dobrado, indo em direção à cadeira

Faça o movimento 10 vezes em cada perna

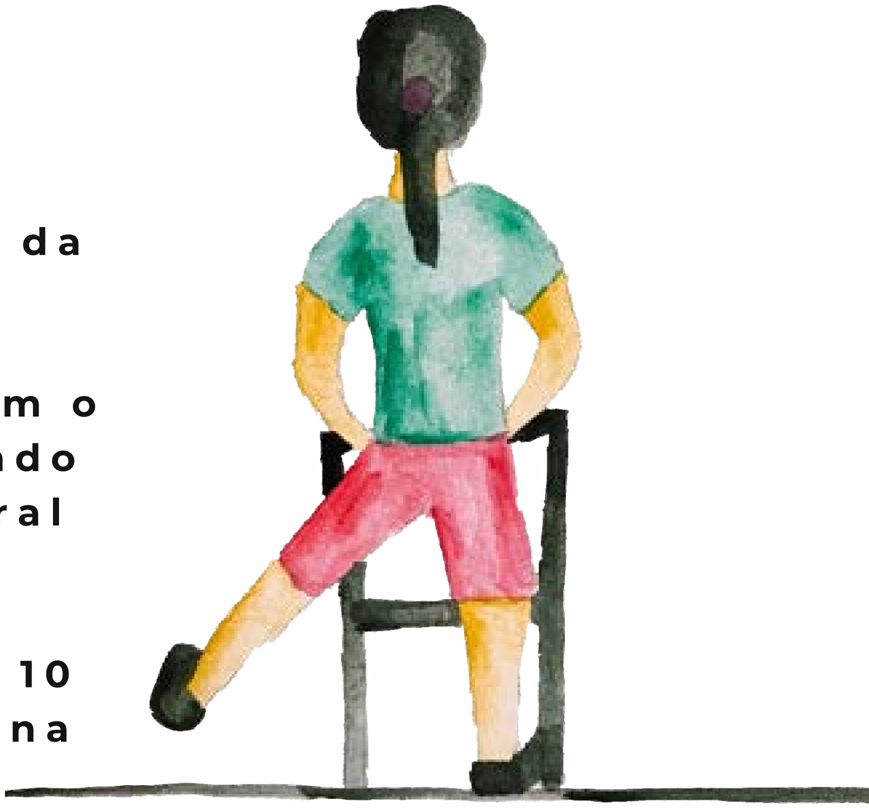

Em pé, atrás da cadeira

Segure no encosto da cadeira

Levante a perna com o joelho esticado, indo em direção à lateral da cadeira

Faça o movimento 10 vezes em cada perna

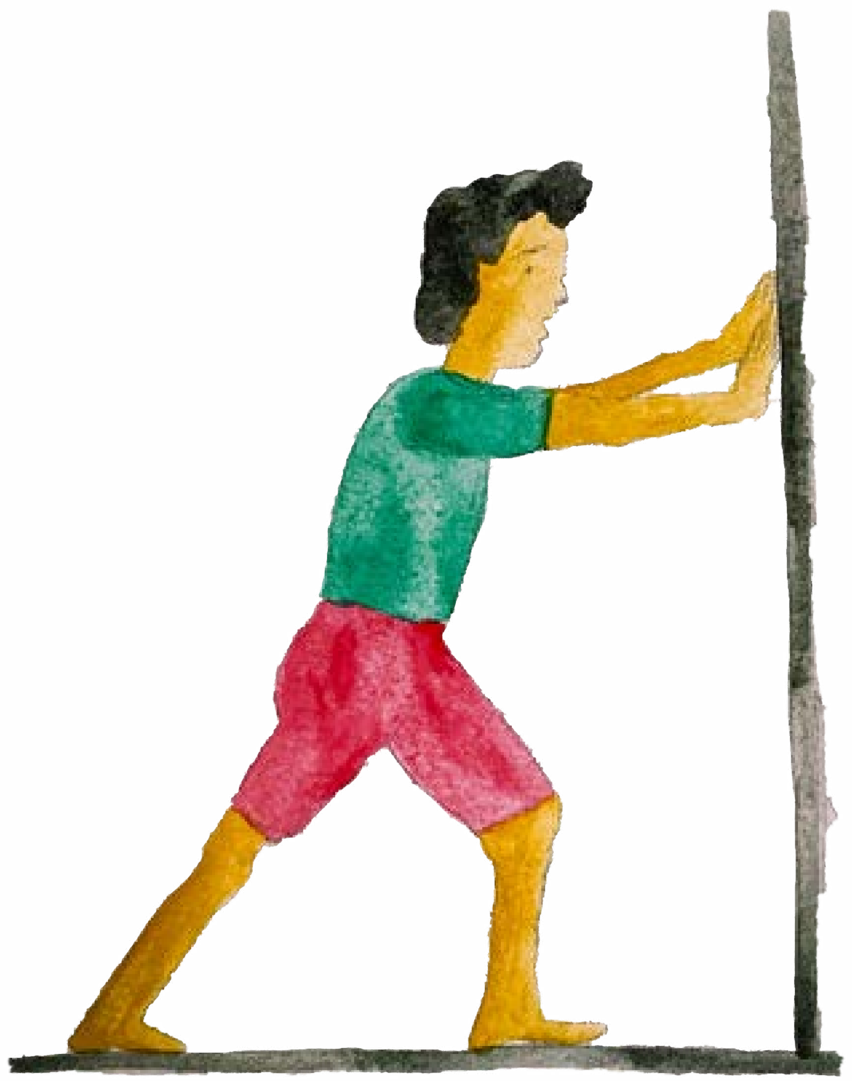

Em pé, fique a frente de uma parede
.
Coloque as duas mãos na parede

Deixe dobre o joelho e de um passo para frente enquanto a outra perna fica esticada e posicionada atrás

Fique nesta posição por 20 segundos

Depois faça a mesma posição com a perna do lado contrário

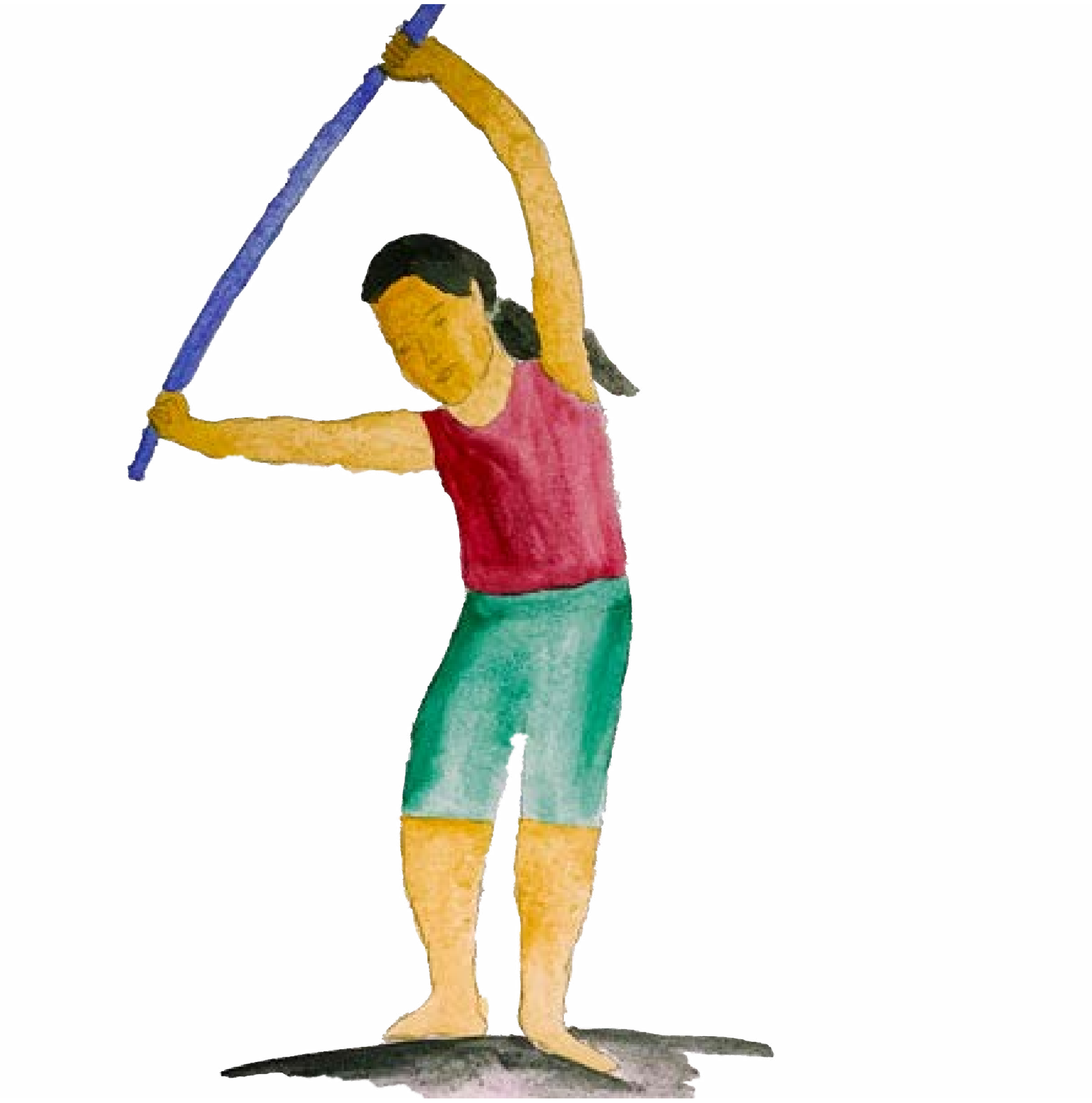

Pegue um cabo de vassoura

Em pé, estique os braços segurando o cabo de vassoura com as duas mãos

Levante os braços até ficar em cima da cabeça

Faça o movimento de levar o cabo de vassoura para o lado direito e esquerdo, contando 10 vezes

Descanse 1 minuto e retorne a fazer o movimento
